# Dodging the Host Interferon-Stimulated Gene Mediated Innate Immunity by HIV-1: A Brief Update on Intrinsic Mechanisms and Counter-Mechanisms

**DOI:** 10.3389/fimmu.2021.716927

**Published:** 2021-07-29

**Authors:** Kumaraswami Chintala, Krishnaveni Mohareer, Sharmistha Banerjee

**Affiliations:** Department of Biochemistry, University of Hyderabad, Hyderabad, India

**Keywords:** HIV-1, restriction factors, PRR, viral counter mechanisms, ISG interferon stimulated genes, PAMP

## Abstract

Host restriction factors affect different phases of a viral life cycle, contributing to innate immunity as the first line of defense against viruses, including HIV-1. These restriction factors are constitutively expressed, but triggered upon infection by interferons. Both pre-integration and post-integration events of the HIV-1 life cycle appear to play distinct roles in the induction of interferon-stimulated genes (ISGs), many of which encode antiviral restriction factors. However, HIV-1 counteracts the mechanisms mediated by these restriction factors through its encoded components. Here, we review the recent findings of pathways that lead to the induction of ISGs, and the mechanisms employed by the restriction factors such as IFITMs, APOBEC3s, MX2, and ISG15 in preventing HIV-1 replication. We also reflect on the current understanding of the counter-mechanisms employed by HIV-1 to evade innate immune responses and overcome host restriction factors. Overall, this mini-review provides recent insights into the HIV-1-host cross talk bridging the understanding between intracellular immunity and research avenues in the field of therapeutic interventions against HIV-1.

## Introduction

Human Immunodeficiency Virus-1 (HIV-1) causes Acquired Immuno Deficiency Syndrome (AIDS), one of the significant contributors to the death of adults worldwide. HIV-1 infection cycle begins with its fusion with the host cell membrane, leading to the release of the viral capsid core into the cytoplasm. The capsid core translocates to the nuclear pore complex by microtubule trafficking, from where it enters into the nucleus. In the nucleus, the capsid core undergoes uncoating, and the reverse-transcribed viral genome integrates into the host genome, forming provirus, allowing transcription and translation of viral genes (1–3). Viral proteins and RNA along with several host proteins, assemble at the plasma membrane and are released as virions. Interferons (IFNs), induced upon viral infection, are the major players in innate defense pathways against any virus. IFN signaling culminates in the transcription of interferon-stimulated genes (ISGs), many of which function as host restriction factors (RFs), which hinder almost every step of the viral life cycle. Thus, IFN signaling is the core of innate defense mechanism against viruses in general. The RFs directly interact with viral components, precluding viral replication, creating an antiviral state in the infected host cell. On the other hand, the evolutionary success of HIV-1 pathogenesis owes to its intricate strategies that block host RFs using its encoded components, such as Vpr (4), Vpu (5), Vif (6), and Nef (7). HIV-1 also targets components of IFN signaling, limiting the expression of RFs. In this mini-review, we deliberate on interferon induced innate mechanisms against HIV-1 infection, exemplified by IFITM (Interferon Induced Transmembrane) proteins, APOBEC3 (**Apo**lipoprotein **B** mRNA **E**diting enzyme **C**atalytic polypeptide like-**3**) proteins, MX2 (Myxovirus resistance-2), and ISG15 (Interferon Stimulated Gene-15) and highlight the current knowledge on the counter-mechanisms by which HIV-1 curtails the induction of innate cascade signaling as well as the restrictions posed by the RFs.

## Innate Antiviral Responses and Intrinsic Restriction of HIV-1 Infection

Pattern Recognition Receptors (PRRs) in the host cell sense Pathogen-Associated Molecular Patterns (PAMPs) to drive IFN-mediated immune responses against viruses including HIV-1 (8). Several PAMPs associated with HIV-1 have been identified, which include capsid-shell, genomic RNA, reverse transcription products such as RNA-DNA hybrids, ssDNA and dsDNA, and proviral transcripts, such as, intron containing viral RNA, suggesting the occurrence of PAMPs during both early (pre-integration) and late steps (post-integration) of HIV-1 replication. These PAMPs are sensed by PRRs/sensors including Toll Like Receptors (TLRs), Retinoic acid Inducible Gene-I Like Receptors (RLRs) and DNA sensors in the HIV-1 infected cells. This section highlights the mechanisms by which host cell senses PAMPs associated with HIV-1 by using different PRRs to activate innate signaling cascade to induce ISGs and the restriction mechanisms mediated by RFs.

### HIV-1 PAMPs and Associated PRRs

During its replication, HIV-1 exposes numerous PAMPs that are recognized by different PRRs present either in the endosomal compartment or cytosol of infected cells. HIV-1 RNA (both genomic RNA and newly synthesized RNA) has been reported to activate RNA-sensors and thus downstream innate signaling cascade.

It is now evident that HIV-1 genomic RNA alone can induce ISGs in peripheral blood mononuclear cells (PBMCs), and macrophages, and that this induction is mediated by cytosolic sensor RIG-I and its adaptor Mitochondrial Associated Viral Sensing protein (MAVS). The determinants of this induction have been identified to be secondary structures of genomic RNA as they were sufficient to induce innate immune responses as full-length genomic RNA (9). In addition to RIG-I, endosome associated PRRs may also act as sensors of HIV-1 genomic RNA. For example, both TLR7 and TLR8 were shown to be required to sense either HIV-1 genomic RNA or its derivatives such as U-rich oligonucleotides in the activation of innate immune signaling (10, 11). Since these studies largely used transfection-based assays to deliver HIV-1 RNA or its oligonucleotide derivatives, it was not clear whether innate immune activation requires virion associated RNA or newly transcribed RNA upon infection. Recent elegant studies conducted in dendritic cells, macrophages, microglia and CD4+T-cells showed that the transcription of viral RNA from provirus and transport of intron-containing RNA (icRNA) mediated by Chromosomal maintenance 1 (CRM1) were required for the induction of ISGs (12–14). These studies further revealed that HIV-1 encoded proteins do not play any role in inducing innate immune signaling. Though, the PRR responsible for the recognition of the newly transcribed icRNA is not yet identified, the adaptor MAVS seems to play an important role in the activation of innate immune signaling mediated by icRNA (13). In addition, abortive transcripts from provirus also seem to induce innate immune cascade. For instance, DEAD box helicase DDX3, a member of RLRs, was shown to bind to abortive viral transcripts and activate antiviral immune response *via* MAVS (15). Therefore, it is suggested that DDX3 or other unidentified RNA sensor(s) may be responsible for MAVS mediated innate immune activation through recognition of icRNA.

As mentioned earlier, the reverse transcription products may act as PAMPs to activate DNA sensors including cyclic GMP-AMP synthase (cGAS) and IFN-γ induced-16 (IFI16). cGAS is a widely studied PRR among the DNA sensors that detects reverse transcripts of HIV-1 (16–18). It was shown that cGAS binds to stem loop structures of ssDNA of HIV-1 in a sequence specific manner, which ultimately results in the activation of innate immune response (19). Although, both reverse transcription and integration of viral DNA seems to be required for the activation of cGAS, the identity of HIV-1 ligand detected by cGAS post integration is not known (17, 18). Similarly, IFI16 is shown to bind to stem loop regions of viral ssDNA and activate the stimulator of interferon gene (STING)-IFN regulatory factor (IRF)-3/7 signaling axis in macrophages (20). Besides, IFI16 senses incomplete HIV-1 reverse transcripts accumulated in the abortively infected CD4+ T-cells and activates caspase-1 resulting in pyroptosis, suggesting the critical role of IFI16 in killing HIV-1 infected cells (21). Taken together, DNA sensors play a crucial role in the innate immune activation against HIV-1. Further, the components present in the viral inoculum *per se* (devoid of HIV-1) also induce ISGs, though not comparable to that of HIV-1 (22). These viral-associated components, primarily extracellular vesicles, seem to activate ISGs transiently, unlike HIV-1, which induces ISG persistently and predominantly. Though IRF1 was shown to be essential for the transient induction of ISGs by extracellular vesicles associated with HIV-1, PRRs for these vesicles and how HIV-1 deals with transiently expressed ISGs are yet to be identified. All the HIV-1 induced innate pathways mediated by PRRs lead to the induction of ISGs that encode antiviral factors, including RFs (23).

#### RFs That Block HIV-1 at Different Steps of Its Life Cycle

RFs induced by IFNs include IFITM proteins, TRIM5α (Tripartite motif-containing protein-5), APOBEC3 proteins, SAMHD1 (SAM and HD domain containing protein 1), MX2, ISG15, SERINC (serine incorporator)-3/5, Schlafen11, ERManI (endoplasmic reticulum α1,2-mannosidase-I), TSPO (Tryptophan-Rich Sensory Protein), ZAP (Zinc-finger antiviral protein), GBP5 (Guanylate binding protein-5) and BST2 (Bone marrow stromal cell antigen-2)/Tetherin (24–26) (**Table 1**). While the mode of antiviral action of several RFs such as IFITMs, MX2, Schlafen11, ZAP, ERManI, GBP5 and ISG15 is known, the associated counter mechanisms by HIV-1 are still not clear. To exemplify, we discuss one representative RF at each stage of HIV-1 infection cycle.

**Table 1:** The antiviral host defense mechanisms and HIV-1 counteractive measures in infected host cells.

S.No	Host factor involved	Host antiviral pathway	HIV-1 modulating factor	HIV-1 imposed counter mechanism	Other viruses	References
**1**	IFITM family	Inhibits fusion of virion with host membrane	Nef	Selective tropic variants resistant to IFITM	Influenza, Measles	(26–28)
**2**	SERINC 3/5	SERINC 3/5 incorporated into virion particles compromises their fusion with new target cells.	Nef	Downregulation of SERINC 5	Several retroviruses (MLV, EIAV)	(28–30)
		Also, they induce differentiation of cells to myeloid lineage				
**3**	SAMHD1	Interferes with RT by degrading cytoplasmic dNTPs	Vpr	Degrades SAMHD1	Other retroviruses including FIV, BIV, MLV, EIAV and M-PMV;vaccinia, Herpes	(28, 31)
**4**	APOBEC3 family	Cytidine deamination during reverse transcription	Vif, Vpr	Degradation of APOBEC3 proteins	Retroviruses, HBV	(28, 32–38)
**5**	RNA-sensor	Activation of ISGs	PR	Modification of viral genome	West Nile virus, Japanese Encephalitis virus, influenza A, Sendai virus, flavivirus, coronaviruses, picornavirus, coxsackievirus B3, chikungunya virus and enterovirus 71	(39–46)
	RIG-I			Degradation of RIG- I		
	RNA or DNA sensor	Activation of ISGs	CA	Cloaking of viral genome		
**6**	DDX3	Activation of ISGs	Gp120	Inhibition of recruitment of TRAF3 to MAVS	Inhibits RT in HBV; HCV and Pox virus	(15, 47, 48)
**7**	TBK-1	Activation of ISGs	Vpr and Vif	Inhibition of TBK1 activation	Several DNA and RNA viruses	(49, 50)
**8**	IRF3	Activation of ISGs	Vif, Vpr and Vpu	Degradation and inhibition of IRF3 localization into the nucleus	Sendai virus, human herpes virus, Newcastle Disease Virus, cytomegalovirus, vaccinia	(51–55)
**9**	MX2	Inhibits nuclear accumulation of viral DNA	Unknown	Mutations in capsid	Myxovirus, herpes viruses,	(28, 56–58)
**10**	Schlafen11	This interferon-stimulated gene may block protein production from non-codon optimized transcripts by binding to tRNA.	CA and other factor?	Unknown	EIAV, flaviviruses, Herpes virus	(28, 59–62)
		Acts at late stage of HIV infection to suppress viral protein production				
**11**	ZAP	The Zn fingers bind to CpG dinucleotides in viral mRNAs causing translational repression	Unknown	Unknown	Retroviruses, alphaviruses, filoviruses, and HBV	(26, 28)
**12**	ISG15	Blocks ubiquitination of viral Gag and Tsg101, affecting virion assembly and release	Unknown	Unknown	CMV, HTLV, influenza, Sindbis, respiratory syncytial virus, dengue and several others	(63–65)
**13**	ERManI/TSPO	Causes misfolding of Gp160	Vpr	Unknown	Influenza virus	(26, 66)

**14**	GBP5	Interferes with the activity of furin (cellular protease) causing defects in processing envelope and incorporation	Unknown	Unknown	MLV, Influenza, Zika, Measles	(28)
**15**	BST2/Tetherin	Virion release	Vpu, Nef	Degradation of tetherin	Retroviruses, herpes- viruses, filo-viruses, VSV and SARS coronavirus	(5, 28, 67, 68)

The table lists the host factors involved in host restriction mechanisms against HIV-1 and the counteractive measures exhibited by HIV-1. HIV-1 either hijacks host machinery or employs its encoded proteins to inhibit or degrade the first line of defenses that include antiviral/restriction factors encoded by ISGs. Other viruses that are restricted by similar host factors are also mentioned. Refer to text for details.

#### Entry

IFITMs mediated antiviral activity ranges from inhibition of viral entry to inhibition of viral protein synthesis, suggesting their broad spectrum of action (27, 69, 70). Specifically, IFITM2 and IFITM3 avert viral entry, while IFITM1 prevents Gag production (70). Further, the IFITMδ20 isoform also causes selective restriction of the tropic variant, X4-virus, which is abundant during the late phase of HIV-1 infection (71).

#### Reverse Transcription**

APOBEC3 family proteins, APOBEC3C, APOBEC3D, APOBEC3F, APOBEC3G, and APOBEC3H, show potent restriction of Vif deficient HIV-1 (72–74). APOBEC3G requires both deaminase-dependent (causing mutations in viral cDNA) and independent mechanisms (attenuating reverse transcriptase activity) to exert its antiviral activity against HIV-1 (75–78). Though APOBEC3F is induced along with APOBEC3G among other ISGs, its antiviral activity does not appear to depend on deaminase catalytic activity to the extent required by APOBEC3G (79) and is packaged into virions (80).

#### Nuclear Entry**

MX2 or MXB, another IFNα-induced ISG reduces nuclear accumulation of viral DNA in the nucleus of infected host cells without affecting the reverse transcription indicating its antiviral role at early stages of HIV-1 infection (56, 57). MX2 also prevents the uncoating of the viral capsid, leading to the abrogation of HIV-1 infection (58). HIV-1-Capsid (CA) mutations that allow alternative entry into the nucleus or abrogating MX2-Capsid interactions make the virus resistant to MX2, suggesting MX2 exerts its antiviral role through interaction with CA (56, 58).

#### Viral Budding and Release**

ISG15 is a ubiquitin-like protein and the highest expressed ISG (81). ISG15 prevents both assembly and release of HIV-1 virions in producer cells (63, 64). Though the ISGylation (conjugation to targets) of HIV-1 viral components is not known, ISG15 blocks ubiquitination of viral Gag and host tumor susceptibility gene Tsg101 (a component of ESCRT-I), affecting virion assembly and release (63). It also disrupts the interaction between VPS4 and LIP5, which is a part of the endosomal sorting complex required for membrane budding and release of HIV-1 virions (64).

Increased expression of RFs upon HIV-1 infection ensures an effective antiviral restriction state in the infected cell; especially when HIV-1 imposes down-regulation of RFs by targeting them for degradation as one of the counter-mechanisms.

## Counter Mechanisms by HIV-1 to Evade Intracellular Intrinsic Immunity

Like other viruses, HIV-1 utilizes its proteins, either directly or through modulation of the host machinery to dodge the restrictions imposed by the innate immune responses. These immune evasion strategies include escaping from being recognized by PRRs to degrading PRRs or their adaptors or RFs. The following section highlights the various counter mechanisms employed by HIV-1 to dodge the innate immune responses involving RFs.

### Escaping Recognition by PRRs: Host-Directed HIV-1 RNA Modification

Host RNA methyltransferases help innate sensors to distinguish endogenous RNA from foreign exogenous RNA by methylating at specific sites on host RNA, including mRNA, tRNA, rRNA, and noncoding RNA. HIV-1 modifies its genomic RNA by exploiting host methyltransferase machinery to escape detection from cytosolic RNA sensors (39–42). It is now evident that the host 2-O-methyltransferase FTSJ3 is recruited with the help of TAR-RNA Binding Protein (TRBP) to HIV-1 genomic RNA. The methylation of HIV-1 RNA prevents innate immune recognition (40). 2-O methylated HIV-1 RNA is not sensed effectively by the cytosolic RNA sensors Melanoma-Differentiation-Associated Gene 5 (MDA5) and RIG-I. In HIV-1-infected dendritic cells and macrophages, the adenosine methylation (N6-methyladenosine) of HIV-1 genomic RNA correlated with resistance against IFN-mediated immune responses (39). The cellular writers, such as methyltransferase-like METTL3 and METTL14, add a methyl group to adenosine and erasers, such as fat mass and obesity-associated protein (FTO) and α-ketoglutarate dependent dioxygenase AlkB homolog-5 (ALKBH5), remove the methyl groups from mRNA. The HIV-1 produced by HEK293T cells overexpressing FTO or ALKBH5 enhanced induction of IFNs in the target cells by decreasing adenosine methylation in its genome and thus preventing the activation of IRF3 and IRF7, suggesting the importance of host-directed HIV-1 RNA modification in the immune evasion strategy (39).

### Cloaking the Viral Genome

Another immune evasion strategy involves cloaking the viral genome through the interaction of HIV-1 CA with host cofactors such as CPSF6 and cyclophilin domain-containing proteins. The CA mutants, N74D and P90A that disrupt the interaction of CA with CPSF6 and cyclophilins, respectively, were unable to replicate in primary monocyte-derived macrophages, unlike wild-type CA (44). This inability to replicate is consistent with increased expression of IFNs and ISGs in the cells infected with HIV-1 CA mutants (N74 and P90A), suggesting that the evading strategy involves interaction between HIV-1 CA and its cofactor(s).

### Degradation/Obstruction of Host Factors Associated With Antiviral Pathways

#### Transcription Factor That Controls Multiple IFN-Inducing Pathways (IRFs)

HIV-1 promotes the degradation of IRF3 as do several other viruses as it is a central transcription factor in the IFN signaling pathway. However, there are contrasting evidence of its mechanism of inhibition of IRF3, promoting efficient viral replication in the target cells, including CD4+T- and myeloid cells. While HIV-1 accessory proteins, Vpr and Vif, degrade IRF3 *via* proteasome pathway (52), HIV-1 Vpu interacts and promotes IRF3 degradation through endolysosome (53). Another study in 293T and Jurkat cell lines argued that Vpu does not colocalize with IRF3 and does not require endolysosome for IRF3 degradation; instead, it involves the activation of caspase-8 to cleave IRF3 (54). The latter study also suggested that Vpr and Vif also mediate caspase-8 dependent IRF3 cleavage. Contrary to that, Manganaro et al. identified that Vpu rather acted as an inhibitor of NFκB, which also results in the activation of ISGs but did not induce degradation of IRF3 and that Vpu played no role in the regulation of IRF3 mediated pathway (82). Recently, Khan et al. demonstrated that HIV-1 Vpr inhibits cGAMP activated IRF3 translocation to the nucleus and promoted the replication of HIV-1 in myeloid cells. They further showed that Vpr localized to the nuclear pore complex, prevented the interaction of karyopherins with IRF3 and NFκB, which is required for their transport into the nucleus. Subsequently, this immune inhibitory mechanism by Vpr was shown to be dependent on the cofactor DCAF1, a substrate receptor of Cullin-4 RING E3-Ubiquitin ligase (CRL4) (55). While most transcription factors are degraded by HIV proteins, Vpu stabilises host ISGs such as p53 that leads the host cell towards apoptosis in the late stage of infection (83).

#### PRR Levels and Their Activity

To further weaken the innate intracellular immunity, HIV-1 also targets PRRs and their adaptors. HIV-1 escapes detection by RIG-I by promoting its degradation in a HIV-1 protease (PR)-dependent manner in monocyte-derived macrophages (43). Surprisingly, protease activity of PR *perse* was not involved in the degradation of this RNA sensor, but the presence of PR was required for the relocalization of RIG-I from cytosol to lysosomes, wherein RIG-I undergoes degradation (43). Further, HIV-1 targets Tank Binding Kinase-1 (TBK1), an adaptor of the IFN signaling pathway. While the ubiquitination of TBK1 was not affected, the phosphorylation of TBK1 required for signal cascade activation was prevented. Accessory proteins, Vpr and Vif have been shown to prevent TBK1 activity and thus downstream signaling (50). Further investigation is required to address how these viral proteins inhibit the autophosphorylation of TBK1.

#### Restriction Factors (RFs)

HIV-1 uses its accessory proteins such as Vif, Vpr, Vpu, and Nef to overcome the restriction imposed by host RFs (**Table 1**). HIV-1 Vif-mediated counter mechanism against APOBEC3G has been the subject of study for the last two decades. It was shown that Vif prevents the antiviral activity of APOBEC3G by promoting its degradation *via* 26s proteasome (32). In addition, later studies suggested that Vif also reduces the levels of APOBEC3G by inhibiting translation of mRNA encoding this protein and transcription of the gene, APOBEC3G, probably by competing with host transcription cofactor CBFβ, which otherwise binds and activates this gene (33, 34). Vif also affects the incorporation and deaminase activity of APOBEC3G, suggesting the multiple ways by which it interferes with RF activities (35–37). In addition, Vpr promotes the degradation of APOBEC3G with the help of its binding protein, VprBP, suggesting a common antiviral pathway affected by two HIV-1 proteins (38). Initial reports regarding the antagonism between HIV-1 Vpu and RF tetherin showed that Vpu binds and promotes internalization of membrane-associated tetherin through their transmembrane interactions, which leads to beta transducing repeat-containing protein (TrCP2) dependent degradation (5). Furthermore, Vpu, through its cytoplasmic domain, is also suggested to displace tetherin from the sites of viral assembly leading to counteracting tetherin-mediated antiviral effect (67). The anti-tetherin role of Vpu has also been associated with IFN resistance by HIV-1 (68).

Serine Incorporator protein 5 (SERINC5) is another potent RF, but not induced by IFN that gets packaged into virions in producer cells and prevents viral fusion with the target cell (29, 30, 84). HIV-1 Nef counteracts this inhibition by promoting SERINC5 trafficking into the endosomal compartment for degradation, thus reducing intracellular SERINC5 in producer cells (29, 30).

## Discussion

**Figure 1:**
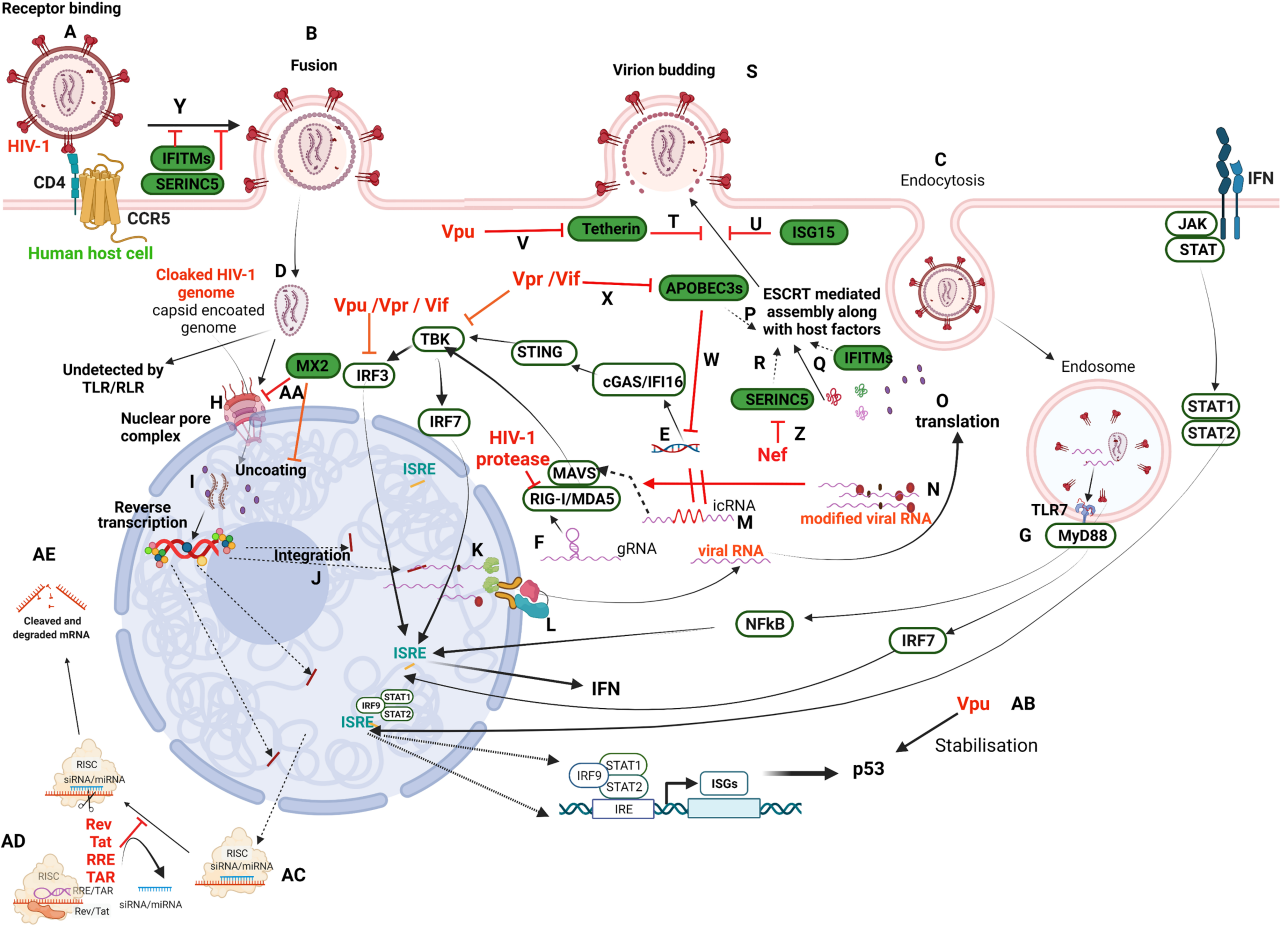
Schematic illustration of HIV-1 infection in a target human host cell and the interception of host restriction pathways by various HIV-1 proteins. HIV-1 infects CD4+ host cells using either of the co-receptors, CCR5 or CXCR4 (here CCR5) **(A)** to fuse with the plasma membrane **(B)** or undergoes receptor-mediated endocytosis **(C)** and various other modes (not in the scope of the present review). Irrespective of the mode of entry, the viral capsid-coated core is released into the cytoplasm **(D)**. Any viral nucleic acids, probably by leakage/disassembly of capsid core, would trigger the pattern recognition receptors present either in the cytosol, such as IFI16 and cGAS **(E)** that recognize viral DNA, or RLRs (RIG-I/MDA5) that recognize viral RNA **(F)**, as well as in endosomes such as TLRs (TLR7) that recognize viral RNA **(G)**. However, the cloaked (coated by capsid) genome escapes recognition by these receptors and enters the nucleus through the nuclear pore complex (NPC) **(H)**. The new school of thought concerning early events of the HIV-1 life cycle is that reverse transcription and uncoating occur in the nucleus **(I)** against the classical theory of these events in the cytoplasm. Once integrated **(J)**, the viral transcripts are made from pro-viral DNA with some incompletely/partially spliced and some completely spliced **(K)**. All the viral transcripts are exported from the nucleus with the hijacked host RNA transport machinery **(L)**. The incompletely spliced intron-containing RNA transcripts (icRNA) are recognized by PRRs in cytosol **(M)** that activate either IRF3 or IRF7 through MAVS. However, the IRF3 activation is inhibited by viral proteins, Vpu/Vpr/Vif. The viral RNA is modified by exploiting the host RNA methyltransferase machinery **(N)** to evade RLR recognition (RIG-I and MDA5). Besides, RIG-I is also targeted by HIV-1 protease. The viral transcripts are translated to produce regulatory proteins (Rev, Tat), accessory proteins (Vpr, Vif, Vpu, Nef) and structural and enzymatic polyproteins (Gag, Pol, Env) **(O)**. The viral proteins and its genome are assembled into budding virions along with host factors that include RFs (APOBEC3s, **(P)**; IFITMs, **(Q)**; SERINC5, **(R)** using host machinery, ESCRT. Viral budding occurs from the host plasma membrane **(S)**, which is targeted by Tetherin **(T)** and ISG15 **(U)**. The viral protein Vpu targets Tetherin **(V)**. APOBEC3s inhibit reverse transcription of viral RNA **(W)** and are targeted by Vif/Vpr **(X)**. IFITM and SERINC5 incorporated into virion inhibit receptor binding and thus fusion **(Y)**. SERINC5 is degraded by Nef **(Z)**. The host factor MX2 inhibits the nuclear import of DNA and uncoating steps in the viral life cycle **(AA)**. Vpu stabilises host ISGs such as p53 that leads to apoptosis of the infected cell **(AB)**. RRE/TAR and Rev/Tat bind to TRBP in the miRNA loaded RISC complex **(AC)** to displace siRNA/miRNA from RISC complex resulting in suppression of RNAi **(AD)** that otherwise leads to activation of RISC signalling and cleavage of transcripts **(AE)**. Refer to text for details. This representative figure reflects several possible pathways and is not intended to reflect the changes in a single cell. Created with BioRender.com

A summary of the various intrinsic mechanisms of host antiviral defense and the counter-mechanisms by HIV is presented through a comprehensive schematic representation (**Figure 1**). The interplay between the host innate intrinsic mechanisms and its counter mechanisms by HIV-1 influences the outcome of HIV-1 infection. RFs play a crucial role in inhibiting the critical steps of HIV-1 replication and thus viral production as part of innate intrinsic mechanism. These RFs are induced along with other antiviral factors during HIV-1 infection upon interaction of PAMPs with host PRRs. Despite the antiviral state created by these mechanisms, HIV-1 counteracts and survives inside the infected cell. To this end, HIV-1 directly targets host RFs for degradation (85) or interferes with innate immune signaling cascade that otherwise induces RFs through Vpr, Vif, Vpu, and Nef or host machinery (**Figure 1**). Furthermore, it is still not clear how HIV-1 evades the restrictions posed by several RFs including MX2 and ISG15. Besides, HIV-1 hijacks host factors that enhance viral productivity, such as Staufen-2 (86), ZNF134 (87); CPSF6 (88–90) Nup153 (91); KIF5B and Nup358 (92). Although certain RFs such as SAMHD1, tetherin, and cholesterol-25-hydroxylase are shown to dampen the innate immune responses, it is not clear whether HIV-1 regulates these RFs for its replication (31, 93, 94). Additionally, it is debated that whether host RNA interference (RNAi) contributes to immune responses against HIV-1. Nevertheless, several miRNAs are differentially regulated in HIV-1 elite controllers as compared to typical progressor patients, suggesting that HIV-1 interferes with host RNAi (95). HIV-1 manipulates host RNAi mechanisms through its RNA elements such as RRE (Rev Response Element) and TAR (Trans Activation Response) as well as Rev and Tat proteins. It was demonstrated that both RRE RNA and TAR elements compete with siRNA to bind to TAR-RNA Binding Protein (TRBP), which is an essential component of the RNA Induced Silencing Complex (RISC), displacing siRNA from TRBP and thus suppressing RNAi (96). On the other hand, the ability of HIV-1 Tat and Rev to suppress host RNAi was traced to the linear Arginine rich motif (ARM) that can bind to RISC loading complex component, TRBP, which otherwise binds to the target mRNA (97). Further studies are required to decipher whether RNAi suppressor activity by HIV-1 contributes to the downmodulation of innate immune responses. Although siRNA is emerging as a powerful therapeutic tool for several viral diseases, in case of HIV, the situation is more complex as it creates a selection pressure for generation of mutants that are resistant to siRNA silencing, apart from its natural tendency to suppress RNAi mechanisms.

An important observation is that not all human cells that allow HIV-1 infection permit viral replication. While host cells such as T-cells and macrophages are a haven for HIV-1 propagation, some non-immune cells such as astrocytes tend to restrict their propagation, allowing the establishment of latency in these reservoir cells. It is possible that cell specific RFs or their level of expression might contribute to the establishment of latency, a major obstacle in the treatment of HIV-1 infected patients. Thus, the studies which reveal the molecular events underlying the restriction mechanisms and factors involved therein, will help in designing strategies to eradicate latency and thus infection in patients.

## Author Contributions

KC and SB conceptualized the manuscript. KC, KM, and SB wrote the paper. KM made the figure. KC, KM, and SB critically analyzed all the information provided. All authors contributed to the article and approved the submitted version.

## Funding

SB acknowledges funding from DBT [BT/HRD/NWBA/38/09/2018 and BT/PR15450/COE/34/46/2016] and DST-SERB for support. KS thanks joint UGC-CSIR JRF, and KM thanks DST for the WOSA fellowship.

## Conflict of Interest

The authors declare that the research was conducted in the absence of any commercial or financial relationships that could be construed as a potential conflict of interest.

## Publisher’s Note

All claims expressed in this article are solely those of the authors and do not necessarily represent those of their affiliated organizations, or those of the publisher, the editors and the reviewers. Any product that may be evaluated in this article, or claim that may be made by its manufacturer, is not guaranteed or endorsed by the publisher.

## Abbreviations

HIV-1, Human Immuno deficiency virus 1; RF, Restriction factor; IFN, Interferon; PRR, Pattern recognition receptor; PAMP, Pathogen associated molecular pattern; cGAS, cyclic GMP-AMP Synthase; IFI16, Interferon γ induced protein 16; STING, Stimulator of interferon gene; IKK, Inhibitor of NFκB kinase; TBK, TANK binding kinase; IRF, Interferon regulatory factor; RLR, Retinoic acid inducible gene I (RIG-I) like receptor; TLR, Toll like receptor; MDA5, Melanoma differentiation associated protein 5; MAVS, Mitochondrial associated viral sensing protein; ISG, Interferon stimulated gene; IRE, Interferon response elements; MX2, Myxovirus resistance protein 2; IFITM, Interferon induced transmembrane protein; APOBEC3, Apolipoprotein B mRNA editing enzyme catalytic polypeptide like 3; SERINC5, Serine incorporator protein 5; Vif, Viral infectivity factor; Vpr, Viral protein R; Vpu, Viral protein U; PR, viral protease; CA, Capsid; TrCP2, beta transducing repeat-containing protein; TRBP, TAR-RNA Binding Protein.

## References

[1] Burdick RC Li C Munshi M Rawson JMO Nagashima K Hu W-S. HIV-1 Uncoats in the Nucleus Near Sites of Integration. Proc Natl Acad Sci (2020) 117:5486–93. 10.1073/PNAS.1920631117 PMC7071919 32094182

[2] Selyutina A Persaud M Lee K KewalRamani V Diaz-Griffero F. Nuclear Import of the HIV-1 Core Precedes Reverse Transcription and Uncoating. Cell Rep (2020) 32:108201. 10.1016/j.celrep.2020.108201 32997983 PMC7871456

[3] Zila V Margiotta E Turoňová B Müller TG Zimmerli CE Mattei S. Cone-Shaped HIV-1 Capsids Are Transported Through Intact Nuclear Pores. Cell (2021) 184:1032–46.e18. 10.1016/j.cell.2021.01.025 33571428 PMC7895898

[4] Arora S Verma S Banerjea AC. HIV-1 Vpr Redirects Host Ubiquitination Pathway. J Virol (2014) 88:9141–52. 10.1128/JVI.00619-14 PMC4136268 24899191

[5] Douglas JL Viswanathan K McCarroll MN Gustin JK Früh K Moses AV. Vpu Directs the Degradation of the Human Immunodeficiency Virus Restriction Factor BST-2/Tetherin *via* a {beta}TrCP-Dependent Mechanism. J Virol (2009) 83:7931–47. 10.1128/JVI.00242-09 PMC2715753 19515779

[6] Feng Y Baig TT Love RP Chelico L. Suppression of APOBEC3-Mediated Restriction of HIV-1 by Vif. Front Microbiol (2014) 5:450. 10.3389/fmicb.2014.00450 25206352 PMC4144255

[7] Shi J Xiong R Zhou T Su P Zhang X Qiu X. HIV-1 Nef Antagonizes SERINC5 Restriction by Downregulation of SERINC5 *via* the Endosome/Lysosome System. J Virol (2018) 92:e00196–18. 10.1128/JVI.00196-18 PMC5952139 29514909

[8] Yin X Langer S Zhang Z Herbert KM Yoh S König R. Sensor Sensibility-HIV-1 and the Innate Immune Response. Cells (2020) 9:254. 10.3390/cells9010254 PMC7016969 31968566

[9] Berg RK Melchjorsen J Rintahaka J Diget E Søby S Horan KA. Genomic HIV RNA Induces Innate Immune Responses Through RIG-I-Dependent Sensing of Secondary-Structured RNA. PloS One (2012) 7:e29291. 10.1371/journal.pone.0029291 22235281 PMC3250430

[10] Meier A Alter G Frahm N Sidhu H Li B Bagchi A. MyD88-Dependent Immune Activation Mediated by Human Immunodeficiency Virus Type 1-Encoded Toll-Like Receptor Ligands. J Virol (2007) 81:8180–91. 10.1128/JVI.00421-07 PMC1951290 17507480

[11] Heil F Hemmi H Hochrein H Ampenberger F Kirschning C Akira S. Species-Specific Recognition of Single-Stranded RNA *via* Toll-Like Receptor 7 and 8. Science (2004) 303:1526–9. 10.1126/science.1093620 14976262

[12] Akiyama H Jalloh S Park S Lei M Mostoslavsky G Gummuluru S. Expression of HIV-1 Intron-Containing RNA in Microglia Induces Inflammatory Responses. J Virol (2020) 95:e01386–20. 10.1128/JVI.01386-20 PMC8092841 33298546

[13] Akiyama H Miller CM Ettinger CR Belkina AC Snyder-Cappione JE Gummuluru S. HIV-1 Intron-Containing RNA Expression Induces Innate Immune Activation and T Cell Dysfunction. Nat Commun (2018) 9:3450. 10.1038/s41467-018-05899-7 30150664 PMC6110775

[14] McCauley SM Kim K Nowosielska A Dauphin A Yurkovetskiy L Diehl WE. Intron-Containing RNA From the HIV-1 Provirus Activates Type I Interferon and Inflammatory Cytokines. Nat Commun (2018) 9:5305. 10.1038/s41467-018-07753-2 30546110 PMC6294009

[15] Gringhuis SI Hertoghs N Kaptein TM Zijlstra-Willems EM Sarrami-Forooshani R Sprokholt JK. HIV-1 Blocks the Signaling Adaptor MAVS to Evade Antiviral Host Defense After Sensing of Abortive HIV-1 RNA by the Host Helicase DDX3. Nat Immunol (2017) 18:225–35. 10.1038/ni.3647 28024153

[16] Sumner RP Harrison L Touizer E Peacock TP Spencer M Zuliani-Alvarez L. Disrupting HIV-1 Capsid Formation Causes cGAS Sensing of Viral DNA. EMBO J (2020) 39:e103958. 10.15252/embj.2019103958 32852081 PMC7560218

[17] Vermeire J Roesch F Sauter D Rua R Hotter D Van Nuffel A. HIV Triggers a cGAS-Dependent, Vpu- and Vpr-Regulated Type I Interferon Response in CD4(+) T Cells. Cell Rep (2016) 17:413–24. 10.1016/j.celrep.2016.09.023 27705790

[18] Gao D Wu J Wu Y-T Du F Aroh C Yan N. Cyclic GMP-AMP Synthase is an Innate Immune Sensor of HIV and Other Retroviruses. Science (2013) 341:903–6. 10.1126/science.1240933 PMC3860819 23929945

[19] Herzner A-M Hagmann CA Goldeck M Wolter S Kübler K Wittmann S. Sequence-Specific Activation of the DNA Sensor cGAS by Y-Form DNA Structures as Found in Primary HIV-1 cDNA. Nat Immunol (2015) 16:1025–33. 10.1038/ni.3267 PMC4669199 26343537

[20] Jakobsen MR Bak RO Andersen A Berg RK Jensen SB Tengchuan J. IFI16 Senses DNA Forms of the Lentiviral Replication Cycle and Controls HIV-1 Replication. Proc Natl Acad Sci USA (2013) 110:E4571–80. 10.1073/pnas.1311669110 PMC3845190 24154727

[21] Monroe KM Yang Z Johnson JR Geng X Doitsh G Krogan NJ. IFI16 DNA Sensor Is Required for Death of Lymphoid CD4 T Cells Abortively Infected With HIV. Science (2014) 343:428–32. 10.1126/science.1243640 PMC3976200 24356113

[22] Nasr N Alshehri AA Wright TK Shahid M Heiner BM Harman AN. Mechanism of Interferon-Stimulated Gene Induction in HIV-1-Infected Macrophages. J Virol (2017) 91:e00744–17. 10.1128/JVI.00744-17 PMC5625512 28768867

[23] Doyle T Goujon C Malim MH. HIV-1 and Interferons: Who’s Interfering With Whom? Nat Rev Microbiol (2015) 13:403–13. 10.1038/nrmicro3449 PMC7768976 25915633

[24] Jia X Zhao Q Xiong Y. HIV Suppression by Host Restriction Factors and Viral Immune Evasion. Curr Opin Struct Biol (2015) 31:106–14. 10.1016/j.sbi.2015.04.004 PMC4476947 25939065

[25] Sandstrom TS Ranganath N Angel JB. Impairment of the Type I Interferon Response by HIV-1: Potential Targets for HIV Eradication. Cytokine Growth Factor Rev (2017) 37:1–16. 10.1016/j.cytogfr.2017.04.004 28455216

[26] Ghimire D Rai M Gaur R. Novel Host Restriction Factors Implicated in HIV-1 Replication. J Gen Virol (2018) 99:435–46. 10.1099/jgv.0.001026 29465030

[27] Lee WYJ Fu RM Liang C Sloan RD. IFITM Proteins Inhibit HIV-1 Protein Synthesis. Sci Rep (2018) 8:14551. 10.1038/s41598-018-32785-5 30266929 PMC6162285

[28] Boso G Kozak CA. Retroviral Restriction Factors and Their Viral Targets: Restriction Strategies and Evolutionary Adaptations. Microorganisms (2020) 8:1965. 10.3390/microorganisms8121965 PMC7764263 33322320

[29] Usami Y Wu Y Göttlinger HG. SERINC3 and SERINC5 Restrict HIV-1 Infectivity and Are Counteracted by Nef. Nature (2015) 526:218–23. 10.1038/nature15400 PMC4600458 26416733

[30] Rosa A Chande A Ziglio S De Sanctis V Bertorelli R Goh SL. HIV-1 Nef Promotes Infection by Excluding SERINC5 From Virion Incorporation. Nature (2015) 526:212–7. 10.1038/nature15399 PMC4861059 26416734

[31] Chen S Bonifati S Qin Z St Gelais C Kodigepalli KM Barrett BS. SAMHD1 Suppresses Innate Immune Responses to Viral Infections and Inflammatory Stimuli by Inhibiting the NF-κB and Interferon Pathways. Proc Natl Acad Sci USA (2018) 115:E3798–807. 10.1073/pnas.1801213115 PMC5910870 29610295

[32] Yu X Yu Y Liu B Luo K Kong W Mao P. Induction of APOBEC3G Ubiquitination and Degradation by an HIV-1 Vif-Cul5-SCF Complex. Science (2003) 302:1056–60. 10.1126/science.1089591 14564014

[33] Stopak K de Noronha C Yonemoto W Greene WC. HIV-1 Vif Blocks the Antiviral Activity of APOBEC3G by Impairing Both its Translation and Intracellular Stability. Mol Cell (2003) 12:591–601. 10.1016/s1097-2765(03)00353-8 14527406

[34] Kim DY Kwon E Hartley PD Crosby DC Mann S Krogan NJ. CBFβ Stabilizes HIV Vif to Counteract APOBEC3 at the Expense of RUNX1 Target Gene Expression. Mol Cell (2013) 49:632–44. 10.1016/j.molcel.2012.12.012 PMC3582769 23333304

[35] Wang Y Kinlock BL Shao Q Turner TM Liu B. HIV-1 Vif Inhibits G to A Hypermutations Catalyzed by Virus-Encapsidated APOBEC3G to Maintain HIV-1 Infectivity. Retrovirology (2014) 11:89. 10.1186/s12977-014-0089-5 25304135 PMC4200127

[36] Feng Y Love RP Chelico L. HIV-1 Viral Infectivity Factor (Vif) Alters Processive Single-Stranded DNA Scanning of the Retroviral Restriction Factor APOBEC3G. J Biol Chem (2013) 288:6083–94. 10.1074/jbc.M112.421875 PMC3585047 23316055

[37] Kao S Khan MA Miyagi E Plishka R Buckler-White A Strebel K. The Human Immunodeficiency Virus Type 1 Vif Protein Reduces Intracellular Expression and Inhibits Packaging of APOBEC3G (CEM15), a Cellular Inhibitor of Virus Infectivity. J Virol (2003) 77:11398–407. 10.1128/jvi.77.21.11398-11407.2003 PMC229358 14557625

[38] Zhou D Wang Y Tokunaga K Huang F Sun B Yang R. The HIV-1 Accessory Protein Vpr Induces the Degradation of the Anti-HIV-1 Agent APOBEC3G Through a VprBP-Mediated Proteasomal Pathway. Virus Res (2015) 195:25–34. 10.1016/j.virusres.2014.08.021 25200749

[39] Chen S Kumar S Espada CE Tirumuru N Cahill MP Hu L. N6-Methyladenosine Modification of HIV-1 RNA Suppresses Type-I Interferon Induction in Differentiated Monocytic Cells and Primary Macrophages. PloS Pathog (2021) 17:e1009421. 10.1371/journal.ppat.1009421 33690734 PMC7984636

[40] Ringeard M Marchand V Decroly E Motorin Y Bennasser Y. FTSJ3 is an RNA 2’-O-Methyltransferase Recruited by HIV to Avoid Innate Immune Sensing. Nature (2019) 565:500–4. 10.1038/s41586-018-0841-4 30626973

[41] Wu L. HIV Evades Immune Surveillance by Methylation of Viral RNA. Biochemistry (2019) 58:1699–700. 10.1021/acs.biochem.9b00152 PMC6445747 30892021

[42] Tirumuru N Zhao BS Lu W Lu Z He C Wu L. N(6)-Methyladenosine of HIV-1 RNA Regulates Viral Infection and HIV-1 Gag Protein Expression. Elife (2016) 5:e15528. 10.7554/eLife.15528 27371828 PMC4961459

[43] Solis M Nakhaei P Jalalirad M Lacoste J Douville R Arguello M. RIG-I-Mediated Antiviral Signaling Is Inhibited in HIV-1 Infection by a Protease-Mediated Sequestration of RIG-I. J Virol (2011) 85:1224–36. 10.1128/JVI.01635-10 PMC3020501 21084468

[44] Rasaiyaah J Tan CP Fletcher AJ Price AJ Blondeau C Hilditch L. HIV-1 Evades Innate Immune Recognition Through Specific Cofactor Recruitment. Nature (2013) 503:402–5. 10.1038/nature12769 PMC3928559 24196705

[45] Rehwinkel J Gack MU. RIG-I-Like Receptors: Their Regulation and Roles in RNA Sensing. Nat Rev Immunol (2020) 20:537–51. 10.1038/s41577-020-0288-3 PMC7094958 32203325

[46] Liu Y Olagnier D Lin R. Host and Viral Modulation of RIG-I-Mediated Antiviral Immunity. Front Immunol (2017) 7:662. 10.3389/fimmu.2016.00662 28096803 PMC5206486

[47] Ko C Lee S Windisch MP Ryu W-S. DDX3 DEAD-Box RNA Helicase is a Host Factor That Restricts Hepatitis B Virus Replication at the Transcriptional Level. J Virol (2014) 88:13689–98. 10.1128/JVI.02035-14 PMC4248967 25231298

[48] Wang H Kim S Ryu W-S. DDX3 DEAD-Box RNA Helicase Inhibits Hepatitis B Virus Reverse Transcription by Incorporation Into Nucleocapsids. J Virol (2009) 83:5815–24. 10.1128/JVI.00011-09 PMC2681949 19297497

[49] Zhao C Zhao W. *TANK*-Binding Kinase 1 as a Novel Therapeutic Target for Viral Diseases. Expert Opin Ther Targets (2019) 23(5):437–46. 10.1080/14728222.2019.1601702 30932713

[50] Harman AN Nasr N Feetham A Galoyan A Alshehri AA Rambukwelle D. HIV Blocks Interferon Induction in Human Dendritic Cells and Macrophages by Dysregulation of TBK1. J Virol (2015) 89:6575–84. 10.1128/JVI.00889-15 PMC4468486 25855743

[51] Schwanke H Stempel M Brinkmann MM. Of Keeping and Tipping the Balance: Host Regulation and Viral Modulation of IRF3-Dependent IFNB1 Expression. Viruses (2020) 12(7):733. 10.3390/v12070733 PMC7411613 32645843

[52] Okumura A Alce T Lubyova B Ezelle H Strebel K Pitha PM. HIV-1 Accessory Proteins VPR and Vif Modulate Antiviral Response by Targeting IRF-3 for Degradation. Virology (2008) 373:85–97. 10.1016/j.virol.2007.10.042 18082865 PMC2312338

[53] Doehle BP Chang K Rustagi A McNevin J McElrath MJ Gale MJ. Vpu Mediates Depletion of Interferon Regulatory Factor 3 During HIV Infection by a Lysosome-Dependent Mechanism. J Virol (2012) 86:8367–74. 10.1128/JVI.00423-12 PMC3421752 22593165

[54] Park SY Waheed AA Zhang Z-R Freed EO Bonifacino JS. HIV-1 Vpu Accessory Protein Induces Caspase-Mediated Cleavage of IRF3 Transcription Factor. J Biol Chem (2014) 289:35102–10. 10.1074/jbc.M114.597062 PMC4271200 25352594

[55] Khan H Sumner RP Rasaiyaah J Tan CP Rodriguez-Plata MT Van Tulleken C. HIV-1 Vpr Antagonizes Innate Immune Activation by Targeting Karyopherin-Mediated NF-κB/IRF3 Nuclear Transport. Elife (2020) 9:e60821. 10.7554/eLife.60821 33300875 PMC7759385

[56] Kane M Yadav SS Bitzegeio J Kutluay SB Zang T Wilson SJ. MX2 is an Interferon-Induced Inhibitor of HIV-1 Infection. Nature (2013) 502:563–6. 10.1038/nature12653 PMC3912734 24121441

[57] Goujon C Moncorgé O Bauby H Doyle T Ward CC Schaller T. Human MX2 is an Interferon-Induced Post-Entry Inhibitor of HIV-1 Infection. Nature (2013) 502:559–62. 10.1038/nature12542 PMC3808269 24048477

[58] Fricke T White TE Schulte B de Souza Aranha Vieira DA Dharan A Campbell EM. MxB Binds to the HIV-1 Core and Prevents the Uncoating Process of HIV-1. Retrovirology (2014) 11:68. 10.1186/s12977-014-0068-x 25123063 PMC4145229

[59] Jakobsen MR Mogensen TH Paludan SR. Caught in Translation: Innate Restriction of HIV mRNA Translation by a Schlafen Family Protein. Cell Res (2013) 23:320–2. 10.1038/cr.2012.155 PMC3587701 23128674

[60] Kim ET Dybas JM Kulej K Reyes ED Price AM Akhtar LN. Comparative Proteomics Identifies Schlafen 5 (SLFN5) as a Herpes Simplex Virus Restriction Factor That Suppresses Viral Transcription. Nat Microbiol (2021) 6:234–45. 10.1038/s41564-020-00826-3 PMC7856100 33432153

[61] Valdez F Salvador J Palermo PM Mohl JE Hanley KA Watts D. Schlafen 11 Restricts Flavivirus Replication. J Virol (2019) 93:e00104–19. 10.1128/JVI.00104-19 PMC6639263 31118262

[62] Lin Y-Z Sun L-K Zhu D-T Hu Z Wang X-F Du C. Equine Schlafen 11 Restricts the Production of Equine Infectious Anemia Virus *via* a Codon Usage-Dependent Mechanism. Virology (2016) 495:112–21. 10.1016/j.virol.2016.04.024 27200480

[63] Okumura A Lu G Pitha-Rowe I Pitha PM. Innate Antiviral Response Targets HIV-1 Release by the Induction of Ubiquitin-Like Protein ISG15. Proc Natl Acad Sci USA (2006) 103:1440–5. 10.1073/pnas.0510518103 PMC1360585 16434471

[64] Pincetic A Kuang Z Seo EJ Leis J. The Interferon-Induced Gene ISG15 Blocks Retrovirus Release From Cells Late in the Budding Process. J Virol (2010) 84:4725–36. 10.1128/JVI.02478-09 PMC2863725 20164219

[65] Ponia BT Arora FEM Kumar É Pegan SD. How ISG15 Combats Viral Infection. Virus Res (2020) 286:198036. 10.1016/j.virusres.2020.198036 32492472 PMC7483349

[66] Beitari S Wang Y Liu SL Liang C. HIV-1 Envelope Glycoprotein at the Interface of Host Restriction and Virus Evasion. Viruses (2019) 11(4):311. 10.3390/v11040311 PMC6521621 30935048

[67] McNatt MW Zang T Bieniasz PD. Vpu Binds Directly to Tetherin and Displaces it From Nascent Virions. PloS Pathog (2013) 9:e1003299. 10.1371/journal.ppat.1003299 23633949 PMC3635990

[68] Kmiec D Iyer SS Stürzel CM Sauter D Hahn BH Kirchhoff F. Vpu-Mediated Counteraction of Tetherin Is a Major Determinant of HIV-1 Interferon Resistance. MBio (2016) 7:e00934–16. 10.1128/mBio.00934-16 PMC4992969 27531907

[69] Compton AA Bruel T Porrot F Mallet A Sachse M Euvrard M. IFITM Proteins Incorporated Into HIV-1 Virions Impair Viral Fusion and Spread. Cell Host Microbe (2014) 16:736–47. 10.1016/j.chom.2014.11.001 PMC7104936 25464829

[70] Lu J Pan Q Rong L He W Liu S-L Liang C. The IFITM Proteins Inhibit HIV-1 Infection. J Virol (2011) 85:2126–37. 10.1128/JVI.01531-10 PMC3067758 21177806

[71] Yandrapally S Mohareer K Arekuti G Vadankula GR. HIV Co-Receptor-Tropism : Cellular and Molecular Events Behind the Enigmatic Co-Receptor Switching. Crit Rev Microbiol (2021) 0:1–18. 10.1080/1040841X.2021.1902941 33900141

[72] Dang Y Wang X Esselman WJ Zheng Y-H. Identification of APOBEC3DE as Another Antiretroviral Factor From the Human APOBEC Family. J Virol (2006) 80:10522–33. 10.1128/JVI.01123-06 PMC1641744 16920826

[73] Hultquist JF Lengyel JA Refsland EW LaRue RS Lackey L Brown WL. Human and Rhesus APOBEC3D, APOBEC3F, APOBEC3G, and APOBEC3H Demonstrate a Conserved Capacity to Restrict Vif-Deficient HIV-1. J Virol (2011) 85:11220–34. 10.1128/JVI.05238-11 PMC3194973 21835787

[74] Jaguva Vasudevan AA Balakrishnan K Gertzen CGW Borvető F Zhang Z Sangwiman A. Loop 1 of APOBEC3C Regulates its Antiviral Activity Against HIV-1. J Mol Biol (2020) 432:6200–27. 10.1016/j.jmb.2020.10.014 33068636

[75] Pollpeter D Parsons M Sobala AE Coxhead S Lang RD Bruns AM. Deep Sequencing of HIV-1 Reverse Transcripts Reveals the Multifaceted Antiviral Functions of APOBEC3G. Nat Microbiol (2018) 3:220–33. 10.1038/s41564-017-0063-9 PMC6014619 29158605

[76] Newman ENC Holmes RK Craig HM Klein KC Lingappa JR Malim MH. Antiviral Function of APOBEC3G Can be Dissociated From Cytidine Deaminase Activity. Curr Biol (2005) 15:166–70. 10.1016/j.cub.2004.12.068 15668174

[77] Iwatani Y Chan DSB Wang F Stewart-Maynard K Sugiura W Gronenborn AM. Deaminase-Independent Inhibition of HIV-1 Reverse Transcription by APOBEC3G. Nucleic Acids Res (2007) 35:7096–108. 10.1093/nar/gkm750 PMC2175344 17942420

[78] Bishop KN Verma M Kim E-Y Wolinsky SM Malim MH. APOBEC3G Inhibits Elongation of HIV-1 Reverse Transcripts. PloS Pathog (2008) 4:e1000231. 10.1371/journal.ppat.1000231 19057663 PMC2584787

[79] Holmes RK Koning FA Bishop KN Malim MH. APOBEC3F can Inhibit the Accumulation of HIV-1 Reverse Transcription Products in the Absence of Hypermutation. Comparisons With APOBEC3G. J Biol Chem (2007) 282:2587–95. 10.1074/jbc.M607298200 17121840

[80] Miyagi E Brown CR Opi S Khan M Goila-Gaur R Kao S. Stably Expressed APOBEC3F has Negligible Antiviral Activity. J Virol (2010) 84:11067–75. 10.1128/JVI.01249-10 PMC2953157 20702622

[81] Mezzaroma I Vullo V Selvaggi C Pierangeli A Turriziani O Gentile M. ISG15 Expression Correlates With HIV-1 Viral Load and With Factors Regulating T Cell Response. Immunobiology (2016) 221:282–90. 10.1016/j.imbio.2015.10.007 26563749

[82] Manganaro L de Castro E Maestre AM Olivieri K García-Sastre A Fernandez-Sesma A. HIV Vpu Interferes With NF-κB Activity But Not With Interferon Regulatory Factor 3. J Virol (2015) 89:9781–90. 10.1128/JVI.01596-15 PMC4577919 26178989

[83] Verma S Ali A Arora S Banerjea AC. Inhibition of β-TrcP-Dependent Ubiquitination of P53 by HIV-1 Vpu Promotes P53-Mediated Apoptosis in Human T Cells. Blood (2011) 117:6600–7. 10.1182/blood-2011-01-333427 21521785

[84] Chen Y-C Sood C Marin M Aaron J Gratton E Salaita K. Super-Resolution Fluorescence Imaging Reveals That Serine Incorporator Protein 5 Inhibits Human Immunodeficiency Virus Fusion by Disrupting Envelope Glycoprotein Clusters. ACS Nano (2020) 14:10929–43. 10.1021/acsnano.0c02699 PMC8274448 32441921

[85] Lata S Mishra R Banerjea AC. Proteasomal Degradation Machinery: Favorite Target of HIV-1 Proteins. Front Microbiol (2018) 9:2738. 10.3389/fmicb.2018.02738 30524389 PMC6262318

[86] Banerjee A Benjamin R Balakrishnan K Ghosh P Banerjee S. Human Protein Staufen-2 Promotes HIV-1 Proliferation by Positively Regulating RNA Export Activity of Viral Protein Rev. Retrovirology (2014) 11:18. 10.1186/1742-4690-11-18 24520823 PMC4016256

[87] Benjamin R Banerjee A Balakrishnan K Sivangala R Gaddam S Banerjee S. Mycobacterial and HIV Infections Up-Regulated Human Zinc Finger Protein 134, a Novel Positive Regulator of HIV-1 LTR Activity and Viral Propagation. PloS One (2014) 9:1–11. 10.1371/journal.pone.0104908 PMC4140746 25144775

[88] Francis AC Marin M Singh PK Achuthan V Prellberg MJ Palermino-Rowland K. HIV-1 Replication Complexes Accumulate in Nuclear Speckles and Integrate Into Speckle-Associated Genomic Domains. Nat Commun (2020) 11:3505. 10.1038/s41467-020-17256-8 32665593 PMC7360574

[89] Achuthan V Perreira JM Sowd GA Puray-Chavez M McDougall WM Paulucci-Holthauzen A. Capsid-CPSF6 Interaction Licenses Nuclear HIV-1 Trafficking to Sites of Viral DNA Integration. Cell Host Microbe (2018) 24:392–404.e8. 10.1016/j.chom.2018.08.002 30173955 PMC6368089

[90] Bejarano DA Peng K Laketa V Börner K Jost KL Lucic B. HIV-1 Nuclear Import in Macrophages is Regulated by CPSF6-Capsid Interactions at the Nuclear Pore Complex. Elife (2019) 8:e41800. 10.7554/eLife.41800 30672737 PMC6400501

[91] Buffone C Martinez-Lopez A Fricke T Opp S Severgnini M Cifola I. Nup153 Unlocks the Nuclear Pore Complex for HIV-1 Nuclear Translocation in Nondividing Cells. J Virol (2018) 92:e41800. 10.1128/JVI.00648-18 PMC6146805 29997211

[92] Dharan A Talley S Tripathi A Mamede JI Majetschak M Hope TJ. KIF5B and Nup358 Cooperatively Mediate the Nuclear Import of HIV-1 During Infection. PloS Pathog (2016) 12:e1005700. 10.1371/journal.ppat.1005700 27327622 PMC4915687

[93] Jin S Tian S Luo M Xie W Liu T Duan T. Tetherin Suppresses Type I Interferon Signaling by Targeting MAVS for NDP52-Mediated Selective Autophagic Degradation in Human Cells. Mol Cell (2017) 68:308–322.e4. 10.1016/j.molcel.2017.09.005 28965816

[94] Wu T Ma F Ma X Jia W Pan E Cheng G. Regulating Innate and Adaptive Immunity for Controlling SIV Infection by 25-Hydroxycholesterol. Front Immunol (2018) 9:2686. 10.3389/fimmu.2018.02686 30524435 PMC6262225

[95] Ayala-Suárez R Díez-Fuertes F Calonge E de la Torre Tarazona HE Gracia-Ruíz de Alda M Capa L. Insight in Mirnome of Long-Term Non-Progressors and Elite Controllers Exposes Potential RNAi Role in Restraining HIV-1 Infection. J Clin Med (2020) 9:2452. 10.3390/jcm9082452 PMC7464121 32751854

[96] Daniels SM Sinck L Ward NJ Melendez-Peña CE Scarborough RJ Azar I. HIV-1 RRE RNA Acts as an RNA Silencing Suppressor by Competing With TRBP-Bound siRNAs. RNA Biol (2015) 12:123–35. 10.1080/15476286.2015.1014759 PMC4615856 25668122

[97] Ponia SS Arora S Kumar B Banerjea AC. Arginine Rich Short Linear Motif of HIV-1 Regulatory Proteins Inhibits Dicer Dependent RNA Interference. Retrovirology (2013) 10:97. 10.1186/1742-4690-10-97 24025624 PMC3848888

